# Supplementary material for: Field-free spin-orbit torque switching via out-of-plane spin-polarization induced by an antiferromagnetic insulator/heavy metal interface
Source: Nat Commun. 2023 May 19;14:2871. doi: 10.1038/s41467-023-38550-1 (PMC10198983; doi:10.1038/s41467-023-38550-1)
Supplement: Supplementary file 1 — Supplementary Information [file 41467_2023_38550_MOESM1_ESM.pdf]

Supplementary Information

of

Field-free spin-orbit torque switching via out-of-plane spin-polarization induced by an antiferromagnetic insulator/heavy metal interface

Mengxi Wang[1, #], Jun Zhou[2, #], Xiaoguang Xu[1, *], Tanzhao Zhang[1], Zhiqiang Zhu[1], Zhixian Guo[1], Yibo Deng[1], Ming Yang[3, *], Kangkang Meng[1], Bin He[4], Jialiang Li[4], Guoqiang Yu[4], Tao Zhu[4], Ang Li[5], Xiaodong Han[5], and Yong Jiang[1,*]

[1]*School of Materials Science and Engineering, University of Science and Technology Beijing, Beijing 100083, China*

[2] *Institute of Materials Research & Engineering, A*STAR, Singapore 138634, Singapore*

[3] *Department of Applied Physics, The Hong Kong Polytechnic University, Hong Kong SAR, China*

[4] *Beijing National Laboratory for Condensed Matter Physics, Institute of Physics, Chinese Academy of Sciences, Beijing 100190, China*

[5] *Faculty of Materials and Manufacturing, Beijing Key Lab of Microstructure and Properties of Advanced Materials, Beijing University of Technology, Beijing 100124, China*

Corresponding authors: Phone: +86-10-6233-4698 / FAX: +86-10-6233-4698,

E-mail: xgxu@ustb.edu.cn (X. G. Xu), kevin.m.yang@polyu.edu.hk (M. Yang), yjiang@ustb.edu.cn (Y. Jiang)

Note 1. The electrical property of NiO

The I-V curve of NiO (20 nm) demonstrates the insulating nature of the NiO layer. This ensures to avoid the current shunting effect in the insulating NiO layer.

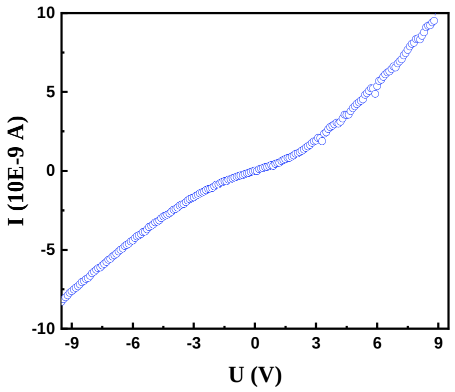

Supplementary Figure 1. The I-V curve of the NiO film.

Note 2. The hysteresis loops of MgO(001)/NiO (20)/Ta (0.2)/Pt (4)/Co (1)/Pt (1) stacks

The hysteresis loops for the in-plane and out-of-plane directions of MgO(001)/NiO (20)/Ta (0.2)/Pt (4)/Co (1)/Pt (1) stacks shows the scenario based on the absence of exchange bias in the MgO/NiO sample. Therefore, the exchange bias between the NiO and Co layers can be excluded in this system via separating the Co and NiO by 4 nm Pt.

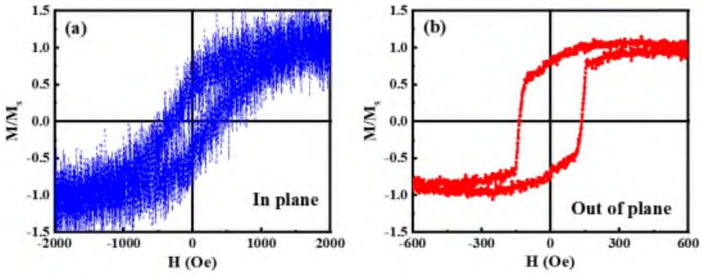

Supplementary Figure 2. The (a) in-plane and (b) out-of-plane magnetic hysteresis loops of the MgO(001)/NiO (20)/Ta (0.2)/Pt (4)/Co (1)/Pt (1) sample.

Note 3. The AHE loop with respect to the magnitude and direction of current

To further probe the source of the signal, we have fabricated another two groups of samples with the stacking structures of NiO (20)/Ta (0.2)/Pt (0.4) and Ta (0.2)/Pt (0.4) deposited on the MgO(001) substrate and measured the R_H-H and R_H-I loops. As shown in Figure S3, we could not observe the AHE-like or SOT-like loop as Figure 2 and Figure 5 in the manuscript. The slight variations of resistance in Figure S3 are mainly caused by the Joule heat effect, which could not contribute the loop-like signal.

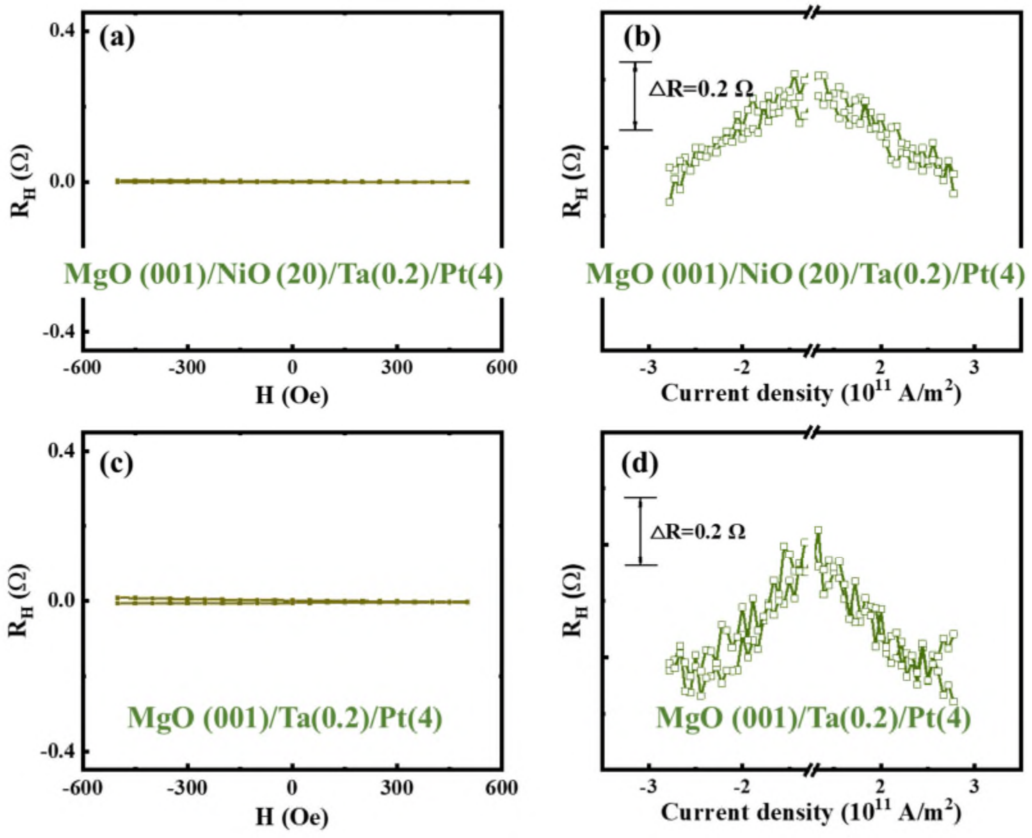

Supplementary Figure 3. The R_H-H (a) and R_H-I (b) loops of sample MgO(001)/NiO (20)/Ta (0.2)/Pt (4); The R_H-H (c) and R_H-I (d) loops of sample MgO(001)/Ta (0.2)/Pt (4).

Note 4. The AHE loop with respect to the magnitude and direction of current

We measured the R-H loops with different current value and directions from 1 mA to 8 mA. The shift of |ΔH(I)| with respect of the magnitude of the applied current is plotted and presented in Figure 4(d).

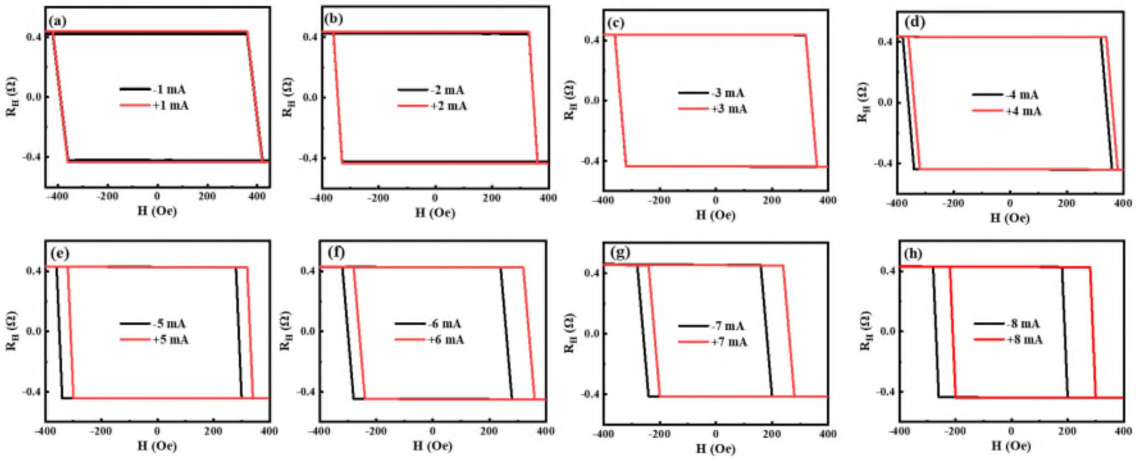

Supplementary Figure 4. The AHE loops with the applied current of (a)-(h) from ± 1 mA to ± 8 mA of the MgO(001)/NiO (20)/Ta (0.2)/Pt (4)/Co (1)/Pt (1) sample.

Note 5. The switching properties of Co with the same thickness for the top and bottom Pt layers

Two samples with the same thickness (4 nm) for the top and bottom Pt layers on MgO(001) and MgO(001)/NiO (20) were fabricated, respectively. The switching properties of Co in the two samples could further prove that the effect of the NiO/HM interface on the modulation of spin polarization.

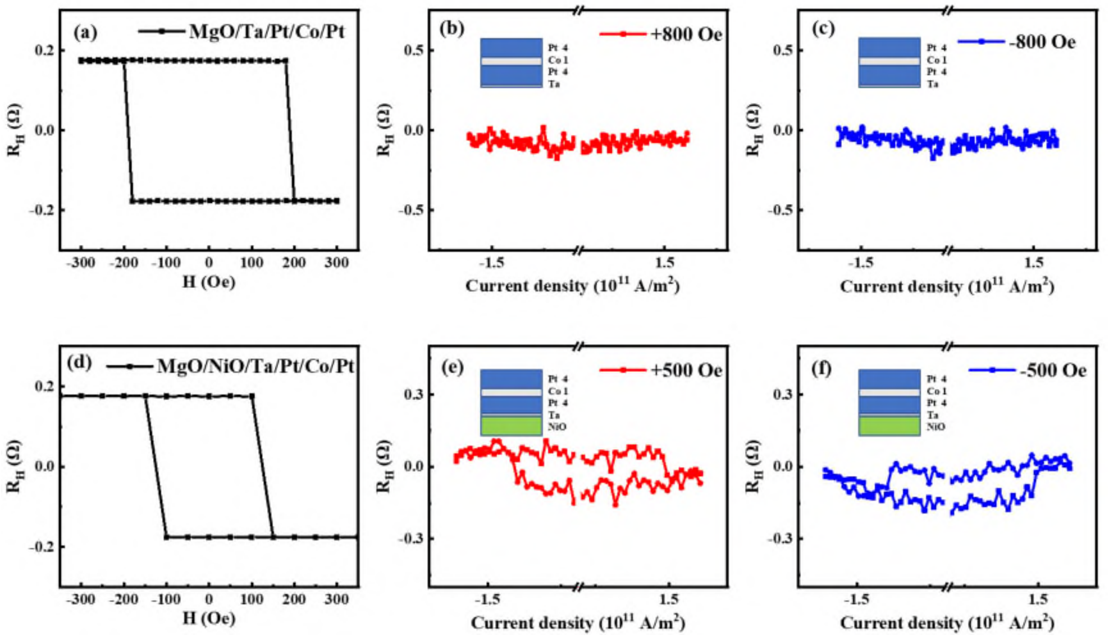

Supplementary Figure 5. (a) The AHE loop of MgO(001)/Ta (0.2)/Pt (4)/Co (1)/Pt (4). (b) and (c) The SOT-based magnetization switching curves of MgO(001)/Ta (0.2)/Pt (4)/Co (1)/Pt (4) under the auxiliary magnetic fields of +800 Oe and -800 Oe, respectively. (d) The AHE loop of MgO(001)/NiO (20)/Ta (0.2)/Pt (4)/Co (1)/Pt (4). (e) and (f) The SOT-based magnetization switching curves of MgO(001)/NiO (20)/Ta (0.2)/Pt (4)/Co (1)/Pt (4) under the auxiliary magnetic fields of +500 Oe and -500 Oe, respectively.

Note 6. The comparison of the roughness for NiO layer growth on different substrates

To reveal the effect of the NiO lattice strain on the spin polarization, we studied the surface roughness of NiO and NiO/Pt films by atomic force microscopy (AFM). The AFM images of the as-deposited NiO and NiO/Pt films on MgO(001) and STO(001) substrates are presented in Figure S6. And the detailed R_q data are summarized in Table S1. It can be seen that the films are smooth with a R_q value lower than 0.2 nm after NiO layer growth and the roughness is also comparable after the metal layers (Ta (0.2)/Pt (4)) deposition.

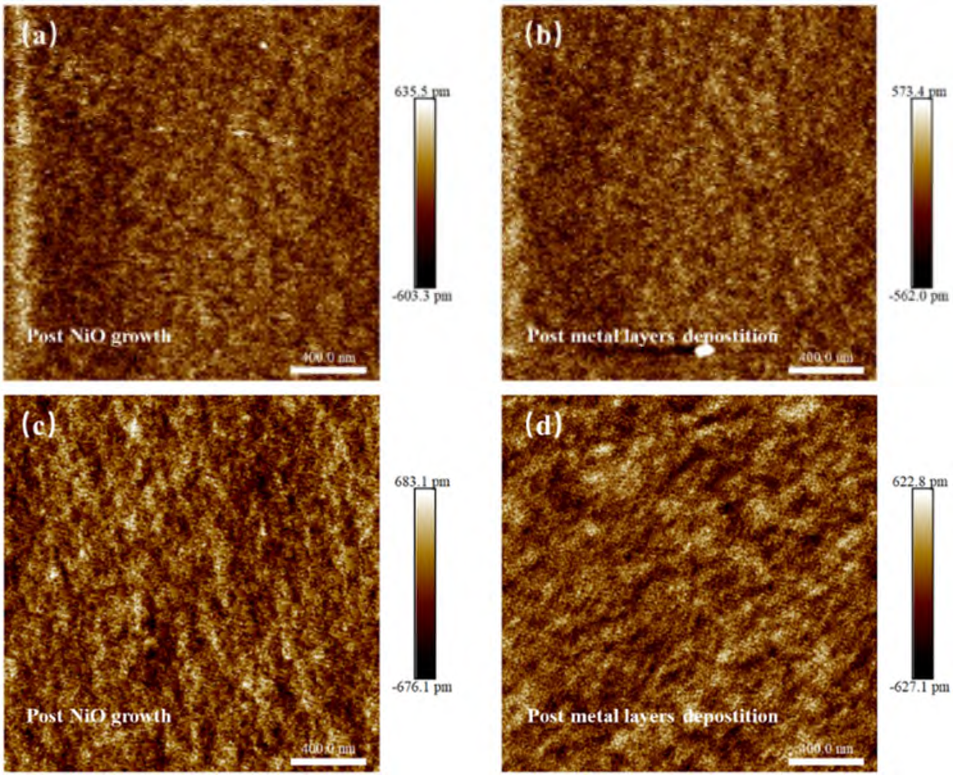

Supplementary Figure 6. The AFM images of (a) MgO/NiO; (b) MgO/NiO/Ta/Pt; (c) STO/NiO and (d) STO/NiO/Ta/Pt.

Samples	Rq (nm)
MgO/NiO	0.176
MgO/NiO/Ta/Pt	0.175
STO/NiO	0.197
STO/NiO/Ta/Pt	0.180

Supplementary Table 1. R_q data of the as-deposited NiO and NiO/Pt films on MgO and STO substrates.

Note 7. The interface quality of the STO(001)/NiO/Pt film.

The crystal quality of the NiO layer and the NiO/Pt interface were characterized by HRTEM as shown in Figure S7.

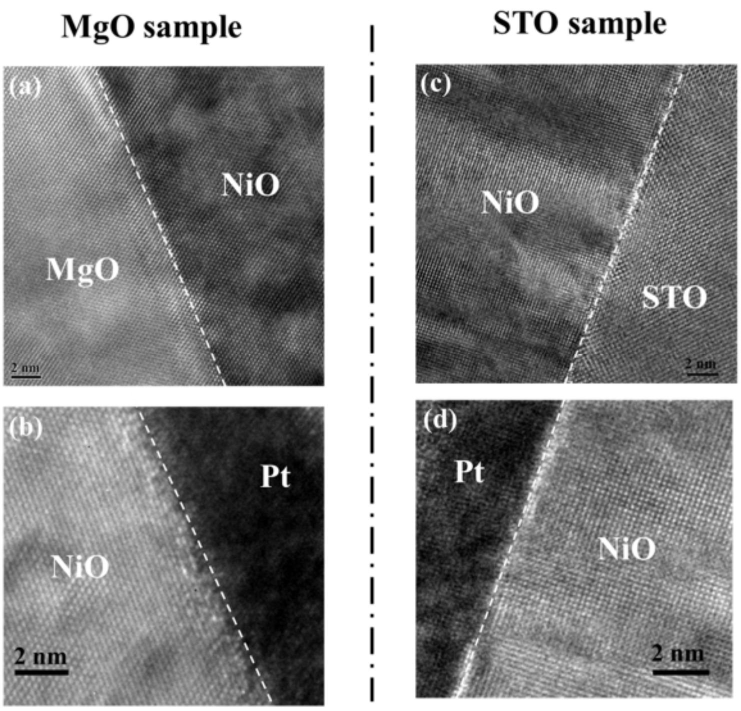

Supplementary Figure 7. HRTEM images of (a) MgO(001)/NiO; (b) NiO/Pt of MgO(001) sample; (c) STO(001)/NiO and (d) NiO/Ta/Pt of STO(001) sample.

Note 8. The switching properties of Co on sample of MgO(111)/NiO (5)/Ta (0.2)/Pt (4)/Co (1)/Pt (1)

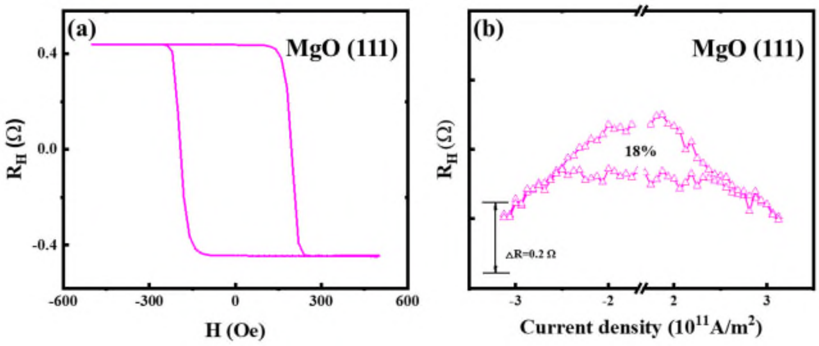

Supplementary Figure 8. (a) The AHE loop and (b) the SOT-based magnetization switching curve of the Hall bar with the stacking structure of MgO(111)/Ta (0.2)/Pt (4)/Co (1)/Pt (1).

Note 9. The magnetic anisotropy energies (MAE) of NiO

The specific magnetization directions examined in this work and their magnetic

anisotropy energies (MAE) are summarized as below.

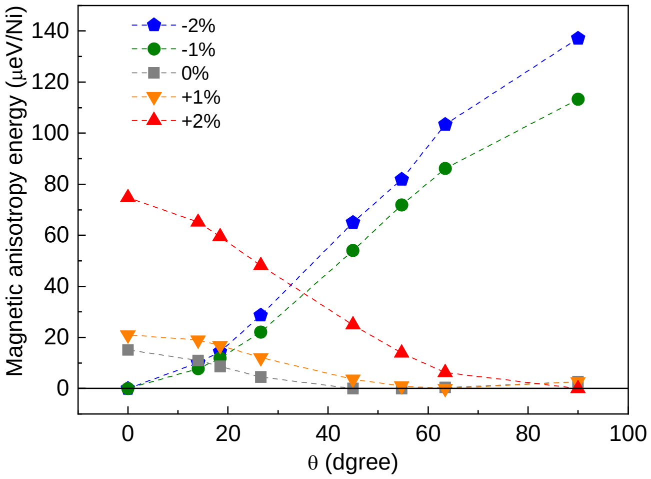

Supplementary Figure 9. The magnetic anisotropy energies (MAE) of NiO in specific magnetization directions under different strains.
